# How mistrust emerged in real time in the Bundibugyo virus disease outbreak: ‘Ebola business’, social media and the history of mistrust in Ebola epidemics in eastern Congo

**DOI:** 10.1136/bmjgh-2026-025522

**Published:** 2026-09-28

**Authors:** Sung Joon Park, Vinh-Kim Nguyen, Freddy Lorima Dz’bo, Emmanuel Bachweki Basia, Kennedy Muhindo Wema, Gloire Mumbesa, Martin Doevenspeck, Jules Villa, Nene Morisho Mwanabiningo

**Affiliations:** 1 Research Group Medical Anthropology, Bernhard Nocht Institute for Tropical Medicine, Hamburg, Germany; 2 Pole Institute, Goma, Democratic Republic of the Congo; 3 Global Health Centre, Graduate Institute of Development Studies, Geneva, Switzerland; 4 Institut Supérieur Pédagogique de Bunia, Bunia, Democratic Republic of the Congo; 5 Bunia Office, Pole Institute, Bunia, Democratic Republic of the Congo; 6 Political Geography, Universität Bayreuth, Bayreuth, Germany; 7 Pasteur Institute, Paris, Île-de-France, France

**Keywords:** Hemorrhagic Fever, Ebola, Public Health, Social Media, Violence

**Summary box**

- In the 2026 Bundibugyo virus disease outbreak in Ituri, eastern Congo, mistrust emerged simultaneously with the public health response, rather than developing after response activities began.
- Communities accept that Ebola is real but interpret case numbers, deaths and response activities through the explanation that ‘Ebola is a business’.
- Memories of corruption, violence, resource extraction and unequal benefit during previous epidemics shape how the current response is understood.
- Social media, including video and AI-generated content, accelerate competing explanations of the outbreak and move rumours rapidly from online platforms into everyday community discussion.
- Preparedness must extend beyond surveillance, vaccines and treatment by building accountable relationships with communities and local organisations before, during and after outbreaks—and by repairing relations damaged by previous responses.

## Introduction

 On 15 May 2026, Congolese health authorities declared a Bundibugyo virus disease (BVD) outbreak in Mongbwalu and Bunia in Ituri Province. BVD is caused by the Bundibugyo virus, one of four orthoebolavirus species that cause Ebola disease in humans. This species is much rarer than Zaire ebolavirus, which has caused most previous Ebola virus disease (EVD) outbreaks. In the months since its declaration, the outbreak has become one of the swiftest on record.^1^ The *pace* of this outbreak raises an old question with new urgency: how could the medical humanitarian apparatus of Ebola response be caught, once again, *unprepared*?^2^ Delayed declaration of this outbreak and the absence of proven treatments and vaccines^3^ are certainly part of the answer.^1^

But viruses do not spread through bodies alone; they spread through histories. The history that matters here is the history of mistrust in the eastern Democratic Republic of the Congo (DRC). There is no commenting on the outbreak without reflecting on this history. Past epidemics, notably the 2014–2016 EVD epidemic in West Africa and the 2018–2020 EVD epidemic in eastern DRC, have repeatedly shown that mistrust drives Ebola’s spread.^4–6^ In the current BVD outbreak, resistance developed *contemporaneously* with the epidemic, marking a significant break from earlier epidemics in which resistance appeared *in response* to efforts to contain them.

This inversion of mistrust also perplexed us. From the first weeks on, we were in the epicentres, Mongbwalu and Bunia, to provide anthropological assistance to the response. As we observed, resistance to the response emerged within days, inundating the hasty construction of response pillars, from surveillance and case management to safe and dignified burials. Makeshift treatment centres were set on fire. The deaths of well-respected community leaders caused fury. Frontline workers were assaulted. And death threats—in Swahili *‘tu uwe yé*’ (‘Let us kill him’)—were repeatedly voiced against humanitarian responders, including ourselves.

The pace of mistrust and resistance may have been baffling. But for researchers who have studied EVD epidemics in eastern Congo and West Africa, have conducted long-term research in eastern Congo or are based in the region, this mistrust is hardly surprising. Suspicion and violence in previous Ebola epidemics foreshadow today’s mistrust. In the 10th EVD epidemic (2018–2020), the response became tainted by naked abuses of power and corruption that fed suspicion, insecurity and increasingly heavy-handed attempts to securitise the epidemic, culminating in a spiral of violence.^5 7^ Memories of past epidemic abuses now resurface in a changed media landscape. Understanding Ebola memories (ie, their underlying cultural logic and the experiences to which they give meaning)^8^ and the architecture of information in the DRC helps explain why mistrust has emerged so quickly.

### Three explanations of unusual deaths influencing mistrust in Ebola outbreaks

Historically, three types of explanations for unusual deaths fuelled mistrust of the public health emergency response. Type 1 explanations associated Ebola with witchcraft, type 2 with haunting and type 3 with financial capture and state failure. From communities’ perspective, all three explanations are reasoned and rest on empirically verifiable phenomena.

In Mongbwalu, a hub for artisanal gold mining in Ituri Province, communities began experiencing an unusual cascade of deaths in March 2026. The deaths were initially attributed to the failure to observe funeral rites for a respected community member. Before the outbreak declaration, the most prevalent explanation was type 2. Type 1 explanations did not gain traction in Ituri, though attributing unusual deaths to powerful, mysterious individuals or disgruntled ancestors is a common and well-known script.^9^

Scholarly debates on mistrust in Ebola epidemics have frequently attributed communities’ denial of Ebola to type 1 and type 2 explanations. But as epidemics progress, these explanations do not withstand popular scrutiny; their scope defies individualistic accounts. No witch or poisoner, people reason, is powerful enough to cause death on this scale.^4^ In Mongbwalu, Rwampara and Bunia, explanations of death shifted quickly after the official declaration. In public fora, discussants asserted that they do not doubt ‘Ebola exists’, and community leaders wasted no time telling communities that a disease, not a spirit, was decimating entire families.

However, this understanding laid the groundwork for the type three explanation. Ebola is real, but it is either an exaggeration or deliberately spread as a clever financial ploy.^10 11^ As one of our interlocutors testified during community outreach activities, “I worked in the 10th epidemic. They said, “Let us tell you a secret, it is a business.” This outbreak is another exaggeration. In eastern Congo we die of all kinds of diseases.” Responders were blamed for having brought the disease and for increasing the number of deaths to make money. Shifts in the number of cases, linked to the challenge of establishing a functioning laboratory for diagnosing BVD, were read as proof that fluctuations in Ebola statistics reflect financial interests. Community leaders and local health workers hesitated to work too closely with the response as communities accused them of having already ‘eaten the money’.

The explanation that ‘Ebola is a business’ echoes the widely shared interpretation of 30 years of armed conflict and insecurity in eastern DRC. Communities and experts alike trace the region’s brutal violence to resource extraction, which profits all conflict parties so long as attacks continue and the conflict remains unsettled and complex.^12^ The apparent parallels between prolonged humanitarian crises caused by Ebola viruses and insecurity fuelled community mistrust and resistance in the 10th EVD epidemic in eastern DRC.^7^ Since 2022, armed conflict has escalated sharply in the region, financed by the extraction of timber, cocoa, gold and other resources.^13^ Unsurprisingly, residents of Mongbwalu and Bunia—shaped by histories of colonial violence and extraction—read official intervention as a front for the informal extortion of rents.^14^

### Information infrastructures in the spread of mistrust

Social media fuel and amplify mistrust. They can be weaponised by algorithmic infrastructures to sow chaos.^15^ In the 2014–2016 epidemic in West Africa and the 2018–2020 epidemic in eastern DRC, smartphones were still relatively rare. Misinformation originated in community conversations, spreading by word of mouth and eventually through Facebook.^16^ By 2018, smartphones and fourth-generation (4G) mobile networks were not as widespread as they are today and were mostly confined to the wealthiest people in the main urban centres. By 2026, they are much more common in eastern DRC, which has also transformed the social architecture of mistrust.^11 17^ Now, directionality is inverted, with misinformation going viral on social media before migrating into communities and into day-to-day conversation. In this sense, social media platforms increasingly function as primary sites of epidemic sense-making rather than merely channels for diffusing information, in effect intensifying and accelerating the development of mistrust.

Today, mistrust is fuelled by a proliferation of videos and AI-generated content, which marks another innovation since 2018.^18^ The content is powerful, resulting in a fog of information requiring WhatsApp group members to continuously ‘demontage’ content, triaging fake from credible news. Obtaining an objective or consensual view of what is really going on is a constant challenge. Different epistemological structures offer competing options for communities to evaluate the credibility of actors and events.^19^

A dramatic consequence of this mistrust is the shunning of public health facilities, out of a mix of fear and defiance. Transmission is driven underground as patients avoid seeking care in specialised facilities, deaths are hidden and funerals are conducted without the necessary precautions. Patients with BVD arrive late at treatment centres with little chance of surviving; the resulting high mortality feeds further mistrust. In hard-to-reach regions, we have heard community leaders tell responders not to come to their communities to take a sick person to the treatment centre, warning that the arrival of their vehicles will escalate fear and anger. The low number of patients discharged from hospitals becomes further proof that mistrust is justified.

## Conclusions

This is the 17th Ebola outbreak in the DRC. Each outbreak has added to a growing body of knowledge about how to contain the next one, including a set of well-tested strategies for building trust: working through respected community leaders; decentralising Ebola care so that it strengthens fragile health systems^20^; staffing the response transparently and representatively; involving young people and demonstrating accountability. These strategies rest on decades of interdisciplinary research integrating epidemiological, behavioural and social science data.^21^ Yet the response was once again caught unprepared and outpaced by mistrust that has spread with unprecedented speed. The acceleration is new. That it happened, given the history of mistrust, is not a surprise.

What can be learnt from this outbreak for building trust? Preparedness must go further and ground interdisciplinary research and response strategies in the biosocial perspective proposed by medical anthropologists.^22^ The biosociality of Ebola epidemics stresses that biological histories of disease transmission and social histories of mistrust constitute each other.^23^ Knowing that history would tell responders what to expect: mistrust that emerges within days and travels through social media. Risk communication and community engagement therefore need to begin in the first weeks, laying the ground for the other pillars before mistrust inundates their establishment. They also need continuous adjustment, whether to new developments such as the rise of social media or to persistent challenges such as armed conflict.

Taking the entanglement of biological and social histories of Ebola epidemics seriously also means continuously extending the response and preparedness of medical humanitarianism. Much can be gained from conflict studies and conflict resolution, well-established fields in eastern Congo, where local organisations in Ituri have spent decades studying how trust and trustworthiness are earned among all parties before negotiations can begin. Supporting researchers and organisations in this field would bring their insights into the history of mistrust to Ebola preparedness and response. Their first lesson concerns time. Building trust takes time, but the rapid onset of mistrust leaves responders without it. Thus, trust must be built before and after outbreaks, not only during them. A second lesson is more difficult for medical-humanitarian responders to accept. Life-saving activities do not speak for themselves as evidence of benevolent intent. Conflict studies have shown this for peacekeeping and for international campaigns to regulate mining in eastern DRC: interventions framed as protection are read within a longer history of conflicts over access to resources.^13 24 25^ Not very differently, life-saving activities in the current outbreak are read within a history of ‘Ebola business’.^7^

Preparedness for future Ebola epidemics, then, cannot be measured by vaccines, treatments and surveillance alone. Each outbreak inherits the trust—or the mistrust—left by the last. Trust is earned slowly, by building relationships. In this sense, preparation is also reparation: repairing the relations damaged by earlier responses is indispensable for preparing for those to come. Building trust before the next virus emerges is not only to anticipate a future emergency but also to answer for a past one.

## Data Availability

All data relevant to the study are included in the article.
